# High-Dose Estradiol-Replacement Therapy Enhances the Renal Vascular Response to Angiotensin II via an AT_2_-Receptor Dependent Mechanism

**DOI:** 10.1155/2015/682745

**Published:** 2015-11-23

**Authors:** Tahereh Safari, Mehdi Nematbakhsh, Roger G. Evans, Kate M. Denton

**Affiliations:** ^1^Department of Physiology, Zahedan University of Medical Sciences, Isfahan, Iran; ^2^Water & Electrolytes Research Center, Isfahan University of Medical Sciences, Isfahan, Iran; ^3^Department of Physiology, Isfahan University of Medical Sciences, Isfahan, Iran; ^4^Isfahan MN Institute of Basic & Applied Sciences Research, Isfahan, Iran; ^5^Department of Physiology, Monash University, Clayton, VIC, Australia

## Abstract

Physiological levels of estrogen appear to enhance angiotensin type 2 receptor- (AT_2_R-) mediated vasodilatation. However, the effects of supraphysiological levels of estrogen, analogous to those achieved with high-dose estrogen replacement therapy in postmenopausal women, remain unknown. Therefore, we pretreated ovariectomized rats with a relatively high dose of estrogen (0.5 mg/kg/week) for two weeks. Subsequently, renal hemodynamic responses to intravenous angiotensin II (Ang II, 30–300 ng/kg/min) were tested under anesthesia, while renal perfusion pressure was held constant. The role of AT_2_R was examined by pretreating groups of rats with PD123319 or its vehicle. Renal blood flow (RBF) decreased in a dose-related manner in response to Ang II. Responses to Ang II were enhanced by pretreatment with estradiol. For example, at 300 ng kg^−1^ min^−1^, Ang II reduced RBF by 45.7 ± 1.9% in estradiol-treated rats but only by 27.3 ± 5.1% in vehicle-treated rats. Pretreatment with PD123319 blunted the response of RBF to Ang II in estradiol-treated rats, so that reductions in RBF were similar to those in rats not treated with estradiol. We conclude that supraphysiological levels of estrogen promote AT_2_R-mediated renal vasoconstriction. This mechanism could potentially contribute to the increased risk of cardiovascular disease associated with hormone replacement therapy using high-dose estrogen.

## 1. Introduction

Women have a lower prevalence of renal and cardiovascular disease than men, at least before menopause [1–5]. The mechanistic basis of sexual dimorphism in the susceptibility to cardiovascular and renal disease remains incompletely understood. However, there is evidence that the renin angiotensin system (RAS) [6, 7] and sex hormones, especially estradiol [1], are critical players.

Angiotensin II (Ang II), the main component of RAS, is of major importance in the regulation of blood pressure, body fluid volume, and electrolyte balance [8]. Even small increases in the plasma concentration of this peptide increase arterial pressure and renal vascular resistance [9]. Ang II also plays an important role in the progression of renal diseases [10, 11]. Activation of the Ang II receptor type 1 (AT_1_R) induces vasoconstriction [7, 12, 13]. For the most part, activation of Ang II receptor type 2 (AT_2_R) has been shown to induce vasodilation [7, 12]. However, there are reports that its activation can induce vasoconstriction, at least in specific vascular beds such as the renal medulla [14, 15].

There is now compelling evidence that estrogen can upregulate AT_2_R function in the systemic and renal vasculature [12]. This action is thought to underlie some of the protection from cardiovascular disease afforded to premenopausal women [16–18]. But such a conclusion is at odds with the observed increase in the incidence of renal [19] and cardiovascular [20] diseases in women taking oral contraceptives. One possible explanation for this paradox relates to the dose of estrogen. That is, while physiological levels of estrogen may blunt Ang II-induced vasoconstriction by upregulating AT_2_R signaling cascades, high-dose estrogen might have the opposite effect or even transform the normal vasodilator influence of AT_2_R activation into a vasoconstrictor action, as has been observed in the renal medulla [14, 15]. To test this hypothesis, in the current study we examined the effects of ovariectomy and hormone “replacement” with a high dose beyond the physiological range, on responses of the renal vasculature of the rat to Ang II* in vivo*. To determine the role of the AT_2_R, rats were studied during treatment with the AT_2_R antagonist PD123319 or its vehicle.

## 2. Methods

### 2.1. Animals

Female Wistar rats (10 to 15 weeks of age) were used in this study (*n* = 28). The rats were housed individually at a temperature of 23–25°C with a 12 h light/dark cycle, with the dark period between 19:00 and 07:00 hours. The rats had free access to water and food. The rats were acclimatized to this diet for at least one week prior to surgery. The experimental procedures were approved in advance by the Isfahan University of Medical Sciences Ethics Committee.

### 2.2. Ovariectomy

The animals were anesthetized with ketamine (75 mg/kg, i.p.). An incision measuring 2 cm in length was made in the subabdominal area. The abdominal muscles were opened and the intestine was retracted. The ureteric tube and the vascular base of ovaries were ligated, and the ovaries were removed. The muscle and skin incisions were closed with sutures and the animals were allowed to recover under a heat lamp. After recovery, the animals were allowed to acclimatize to the regular diet for one week. Then, they were randomly divided into four experimental groups. Two groups (*n* = 5 each) received 0.5 mg/kg/week estradiol valerate (Aburaihan Co., Tehran, Iran) in sesame oil via intramuscular injections for two weeks. Two groups (*n* = 5 each) received the sesame oil only. At the end of the two-week run-in period, a terminal experiment was performed under general anesthesia, during which groups of estradiol and vehicle-treated rats were treated with the AT_2_R antagonist PD123319 or its vehicle, and renal vascular responses to Ang II were examined (see below). A fifth group (*n* = 8) was sham operated. These animals were not subjected to the terminal experiment, but body weight and uterine weight were determined two weeks after surgery to allow comparison with the other experimental groups.

### 2.3. Terminal Procedures

Rats were anesthetized (Inactin; thiobutabarbital, 175 mg kg^−1^ i.p.; Sigma, St. Louis, MO, USA) and the trachea was isolated and cannulated to facilitate ventilation. Catheters were implanted into the jugular vein and the carotid and femoral arteries. Mean arterial pressure (MAP) was measured from the carotid artery. Femoral arterial pressure was considered as the renal perfusion pressure (RPP). In order to maintain this pressure at control levels (to avoid the direct effect of RPP elevation induced by Ang II administration) during infusion of Ang II, an adjustable clamp was placed around the aorta above the level of the renal arteries. The left kidney was placed in a stable cup. A flow probe (type 2SB; Transonic Systems, Ithaca, NY, USA) was placed around the renal artery, so that left kidney renal blood flow (RBF) could be monitored by transit-time ultrasound flowmetry. Body temperature was continuously monitored throughout the experiment. Experimental manipulations commenced 30–60 minutes after completion of the surgical preparation. MAP, RPP, and RBF were measured continuously throughout the experiment as 2-second averages, via a data acquisition system. Renal vascular resistance was calculated as MAP/RBF.

### 2.4. Experimental Protocol

Groups of ovariectomized female rats and ovariectomized rats treated with high-dose estradiol received either the AT_2_R antagonist PD123319 (1 mg kg^−1^ plus 1 mg kg^−1^ h^−1^ from stock of 0.5 mg/mL) or its vehicle (2 mL kg^−1^ plus 2 mL kg^−1^ h^−1^ 154 mmol L^−1^ NaCl) intravenously. This dose of PD123319 was similar to previous studies [21–23], and it was selected based on Macari et al.'s report that PD123319 is highly selective for AT2R at doses less than 1000 *μ*g/kg/min [24]. The antagonist infusions continued for the whole experiment. Thirty minutes after commencing the infusion of PD123319 or its vehicle, a series of intravenous (via the jugular vein) infusions of Ang II (0, 30, 100, and 300 ng kg^−1^ min^−1^) commenced in all rats. Each dose was administered until equilibration of arterial blood pressure was achieved (about 10 min), and then the measurements were performed for 3–5 minutes. The rats were killed by overdose of anesthetic at the end of the experiments, and the kidney and uterus were removed and weighted.

### 2.5. Statistical Analysis

Data are expressed as mean ± SEM. One-way analysis of variance (ANOVA) was applied to baseline data. Between-groups comparisons were then made using Tukey's test. Repeated measures ANOVA was used to determine whether the responses to Ang II were altered by estrogen therapy or PD123319 or an interaction between these two treatments. The Greenhouse-Geisser correction was applied to *P* values derived from within-subjects factors [25]. Two-tailed *P* ≤ 0.05 was considered statistically significant.

## 3. Results

### 3.1. Baseline Measurements

**Figure 1:**
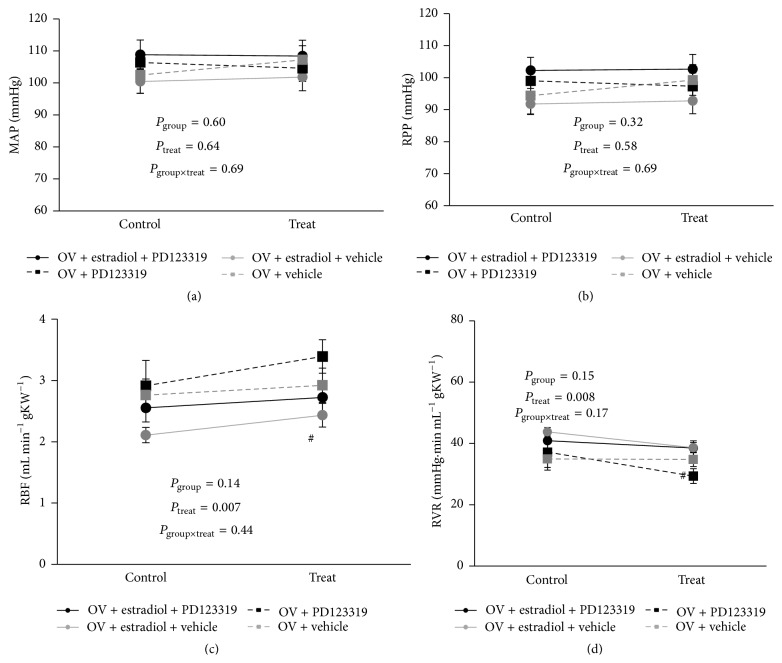
Hemodynamic variables before and after administration of vehicle or PD123319. Data are presented as mean ± SEM. The *P* values were derived from repeated measures ANOVA with factor groups, treatment, and their interaction. *n* = 5 per group. MAP, mean arterial pressure; RPP, renal perfusion pressure; RBF, renal blood flow per gram kidney weight; RVR, renal vascular resistance; OV, ovariectomized group.

No significant differences were observed between the groups with respect to body weight, kidney weight, MAP, RPP, RBF, and RVR. However, uterine weight was 5-fold greater in estradiol-treated animals compared to vehicle-treated animals (Table 1 and Figure 1). In addition, the uterine weight of sham operated rats was 2.7-fold greater than that of the vehicle-treated ovariectomized rats but 47% less than in the estradiol-treated rats (Table 1). Collectively, these observations indicate that the dose of estradiol we used was supraphysiological.

### 3.2. Responses to PD123319 and Its Vehicle

There was little or no change in MAP after treatment with PD123319 or its vehicle commenced (Figure 1). Across all 20 rats, RBF increased by 12.6 ± 3.5% and RVR reduced by 8.5 ± 2.8% after administration of either PD123319 or its vehicle. However, these responses were indistinguishable in rats treated with PD123319 relative to those treated with its vehicle. Thus, it appears that renal vasodilatation occurred over time during the experiment, independent of whether rats were treated with PD123319 or its vehicle.

### 3.3. Responses to Ang II Infusion

**Figure 2:**
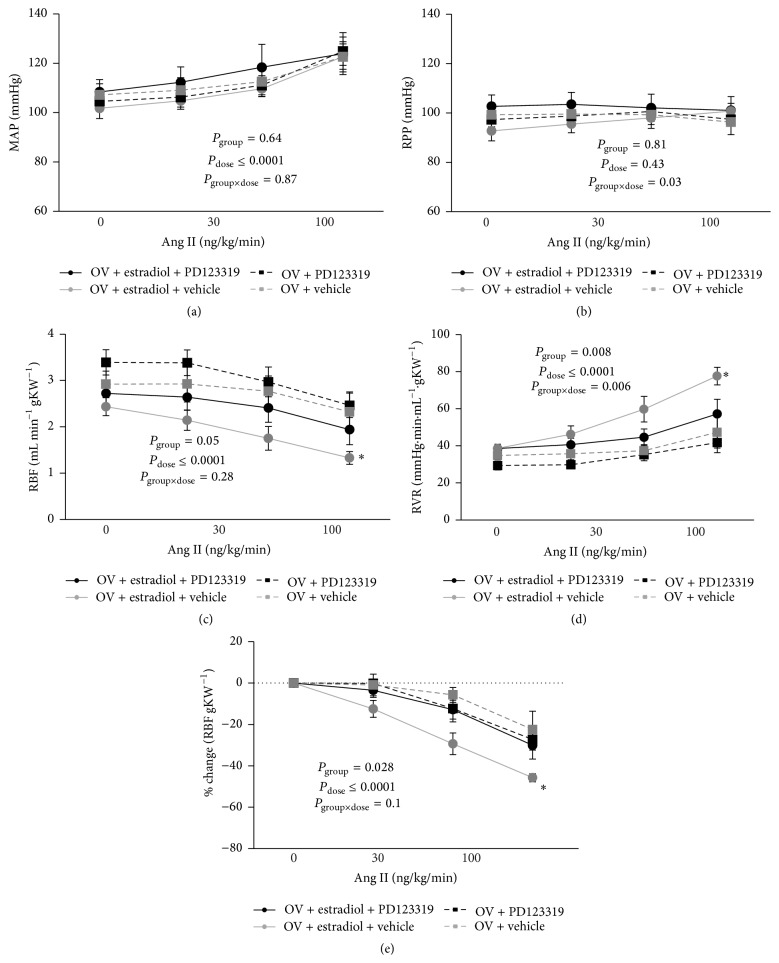
Effects of vehicle or PD123319, on responses to Ang II infusion. Data are shown as mean ± SEM. The data for RBF are also presented as percentage change from baseline. *P* values were derived from repeated measures ANOVA with factors group, dose (of Ang II), and their interaction. *∗* represents *P* ≤ 0.05 for comparison of the response to 300 ng/kg/min in ovariectomized rats treated with estradiol and the vehicle for PD123319 compared with all other groups, derived from Tukey's* post hoc* test. *n* = 5 per group. MAP, mean arterial pressure; RPP, renal perfusion pressure; RBF, renal blood flow per gram kidney weight; RVR, renal vascular resistance; OV, ovariectomized group.

Ang II infusion resulted in dose-related increases in MAP in female rats (Figure 2). The increases in MAP in response to graded doses of Ang II infusion were not significantly altered by pretreatment with either estradiol or PD123319. However, in all groups, RPP was kept relatively constant during Ang II infusion by manipulation of the aortic clamp (Figure 2).

RBF decreased and RVR increased in a dose-related fashion in response to infusion of Ang II (Figure 2; *P*_dose_ < 0.0001). In ovariectomized rats pretreated with the vehicle for estradiol, responses to Ang II appeared to be little affected by PD123319. For example, 300 ng kg^−1^ min^−1^ Ang II reduced RBF by 27 ± 5% and increased RVR by 42 ± 14% in rats pretreated with the vehicle for estradiol and then treated with PD123319 and reduced RBF by 23 ± 9% and increased RVR by 36 ± 23% in rats pretreated with the vehicle for estradiol and then treated with the vehicle for PD123319 (Figure 2). The greatest response to Ang II was observed in ovariectomized rats treated with estradiol but not PD123319. For example, 300 ng kg^−1^ min^−1^ Ang II reduced RBF by 46 ± 2% and increased RVR by 101 ± 7% in this group (Figure 2). In contrast, responses of RBF to Ang II in rats pretreated with estradiol and then treated with PD123319 were similar to those of the two groups that were not treated with estradiol. For example, 300 ng kg^−1^ min^−1^ Ang II reduced RBF by 30 ± 7% and increased RVR by 46 ± 14% in this group (Figure 2).

## 4. Discussion

This study was designed to determine the acute RBF response to Ang II infusion in the presence of fixed RPP in ovariectomized rats treated with supraphysiological dose of estradiol. The major new finding of the current study was that the renal vasoconstrictor response to Ang II in ovariectomized rats was enhanced by high-dose estradiol pretreatment. Interestingly, this enhanced response was not observed when AT_2_R were acutely blocked with PD123319. Taken together with previous observations in the literature, discussed in detail below, our current observations suggest that the impact of estrogen on AT_2_R function may be more complex than previously thought. That is, while physiological levels of estrogen might promote the vasodilator actions of AT_2_R activation in the renal vasculature, supraphysiological levels might instead promote vasoconstriction. It is tempting to speculate that such a phenomenon might underlie, at least in part, the apparently deleterious effects of high-dose estrogen therapy on risk of cardiovascular and renal disease in postmenopausal women.

It is generally regarded that AT_2_R, located on endothelial cells, predominately mediates vasodilatation via the generation of nitric oxide and as such opposes the vasoconstrictor actions driven by the AT_1_R [26, 27]. However, AT_2_R-mediated vasoconstriction has been observed under a variety of conditions, including in the mesenteric vasculature of spontaneously hypertensive rats (SHR)* in vitro* [28], the cerebral vasculature during hemorrhage in rats* in vivo* [29], the rat hydronephrotic kidney [30], the kidneys of rats with heart failure [31], and the renal medullary circulation of both normal rats and rabbits [15] and rats with renovascular hypertension [14]. The AT_2_R also appears to mediate ~20% of Ang II-induced vasoconstriction in SHR during development of hypertension [32]. It is suggested that AT_2_R-mediated vasoconstriction is due to an increase in smooth muscle cell AT_2_R expression [26]. Our current findings indicate that supraphysiological levels of estrogen are also able to promote the vasoconstrictor actions of AT_2_R activation.

In contrast to our current findings, there is considerable evidence that physiological levels of estrogen promote the vasodilator action of AT_2_R. For example, a lower AT_1_R/AT_2_R ratio was found in female as compared to male SHR and this was associated with a lower arterial pressure in the females [33]. Also, it has been demonstrated that low dose Ang II decreases arterial pressure in females via AT_2_R activation [34] and that this effect was abolished by ovariectomy and restored by estrogen replacement [35]. In addition, it has been demonstrated that the attenuated pressor response to chronic Ang II infusion observed in female mice is abolished in estrogen receptor alpha knockout mice [36] and in aged reproductively senescent mice [37]. Evidence also suggests that arterial pressure is kept normal during pregnancy by a decreased vascular responsiveness to Ang II modulated in part by upregulation of AT_2_R expression. This was demonstrated in AT_2_R null mice in which arterial pressure rose significantly during pregnancy [38]. Finally, Ang II caused dose-dependent forearm vasodilatation in female patients following 3-week candesartan treatment; and PD123319 infusion elevated baseline forearm vascular resistance, suggesting that tonic AT_2_R-mediated vasodilatation contributes to the hemodynamic profile of AT_1_R blockade [39]. Thus, there appears to be a complex relationship between the bioavailability of estrogen and the regulation of AT_2_R function.

A number of limitations of our current study should be acknowledged. Firstly, we did not assess the impact of estrogen therapy on the expression of angiotensin receptors in the kidney. Secondly, we did not investigate the mechanisms underlying AT_2_R-mediated renal vasoconstriction, which remain unknown [24]. Thus, the precise mechanisms that underlie the complex dose-response relationship for estrogen, which allow physiological levels to promote AT_2_R-mediated renal vasodilation and high levels to promote AT_2_R-mediated vasoconstriction, must be the subject of future studies. However, our study also has a number of strengths. Firstly, we can be confident that the dose of estradiol we used was supraphysiological, since it resulted in marked hypertrophy of the uterus. Secondly, we can be confident that the dose of PD123319 used was sufficient to block AT_2_R in the kidney, since we have previously shown this dose to abolish AT_2_R-mediated vasoconstriction in the renal medullary circulation of rats [14].

In conclusion, our current findings indicate that supraphysiological levels of estrogen can promote AT_2_R-mediated vasoconstriction. This action could potentially underlie some of the detrimental influence of high-dose estrogen replacement therapy on the risk of cardiovascular disease.

## Figures and Tables

**Table 1:** Hemodynamic variables before vehicle or PD123319 administration and body, uterus, and kidney weights at postmortem.

Group	BW g	UW mg	KW g	MAP mmHg	RPP mmHg	RBF mL/min/g KW	RVR mmHg/mL/min/g KW
OV	192 ± 8	35 ± 4	0.66 ± 0.03	103 ± 6	94 ± 6	2.7 ± 0.3	35 ± 3
OV + PD	203 ± 11	45 ± 12	0.70 ± 0.03	106 ± 2	99 ± 2	2.9 ± 0.4	37 ± 6
OV + E	185 ± 9	202 ± 19^*∗*^	0.62 ± 0.03	100 ± 4	91.7 ± 3	2.1 ± 0.1	44 ± 1
OV + E + PD	183 ± 6	201 ± 26^*∗*^	0.72 ± 0.04	109 ± 5	102 ± 4	2.5 ± 0.2	44 ± 2
Sham	190 ± 4	107 ± 7^#^		—	—	—	—

*P*_ANOVA_	0.4	<0.0001	0.2	0.5	0.3	0.2	0.3

Data are presented as mean ± SEM of *n* = 5. The *P* values were derived from one-way ANOVA. Specific contrasts were generated by Tukey's *post hoc* comparisons. ^*∗*^ *P* ≤ 0.05 for comparison with ovariectomized rats treated with the vehicles for estrogen and PD123319. ^#^ *P* ≤ 0.05 for comparison with all ovariectomized rats. OV: ovariectomized, E: estradiol, PD: PD123319, BW: body weight, UW: uterus weight, KW: kidney weight, MAP: mean arterial pressure, RPP: renal perfusion pressure, RBF: renal blood flow per gram kidney weight, and RVR: renal vascular resistance.
